# Do physiological and pathological stresses produce different changes in heart rate variability?

**DOI:** 10.3389/fphys.2013.00197

**Published:** 2013-07-30

**Authors:** Andrea Bravi, Geoffrey Green, Christophe Herry, Heather E. Wright, André Longtin, Glen P. Kenny, Andrew J. E. Seely

**Affiliations:** ^1^Department of Cellular and Molecular Medicine, University of OttawaOttawa, ON, Canada; ^2^Ottawa Hospital Research InstituteOttawa, ON, Canada; ^3^Therapeutic Monitoring Systems Inc.Ottawa, ON, Canada; ^4^Human and Environmental Physiology Research Unit, School of Human Kinetics, University of OttawaOttawa, ON, Canada; ^5^Department of Physics, University of OttawaOttawa, ON, Canada; ^6^Departments of Surgery and Critical Care Medicine, University of OttawaOttawa, ON, Canada

**Keywords:** dimensions of variability, domains of variability, exercise, physical activity, disease, sepsis

## Abstract

Although physiological (e.g., exercise) and pathological (e.g., infection) stress affecting the cardiovascular system have both been documented to be associated with a reduction in overall heart rate variability (HRV), it remains unclear if loss of HRV is ubiquitously similar across different domains of variability analysis or if distinct patterns of altered HRV exist depending on the stressor. Using Continuous Individualized Multiorgan Variability Analysis (CIMVA™) software, heart rate (HR) and four selected measures of variability were measured over time (windowed analysis) from two datasets, a set (*n* = 13) of patients who developed systemic infection (i.e., sepsis) after bone marrow transplant (BMT), and a matched set of healthy subjects undergoing physical exercise under controlled conditions. HR and the four HRV measures showed similar trends in both sepsis and exercise. The comparison through Wilcoxon sign-rank test of the levels of variability at baseline and during the stress (i.e., exercise or after days of sepsis development) showed similar changes, except for LF/HF, ratio of power at low (LF) and high (HF) frequencies (associated with sympathovagal modulation), which was affected by exercise but did not show any change during sepsis. Furthermore, HRV measures during sepsis showed a lower level of correlation with each other, as compared to HRV during exercise. In conclusion, this exploratory study highlights similar responses during both exercise and infection, with differences in terms of correlation and inter-subject fluctuations, whose physiologic significance merits further investigation.

## Introduction

Several studies have shown that heart rate variability (HRV) can be used to characterize physiological (e.g., physical exercise, heat and cold stress), as well as pathological stress affecting the cardiovascular system. The utility of HRV in identifying illness states has been discussed in multiple reviews, and specific applications include the classification of heart failure, the prediction of mortality after myocardial infarction, and the estimation of autonomic modulation (Task Force, 1996; Seely and Macklem, 2004; Huikuri et al., 2009; Bravi et al., 2011). Similarly for physiological stress, measurement of HRV during physical exercise has been shown to estimate autonomic modulation, assess the level of fitness, characterize the beneficial effects of physical exercise, and many other applications (Perini and Veicsteinas, 2003; Lewis and Short, 2010; Routledge et al., 2010).

One of the major findings extracted from this collection of results is that when the cardiovascular system is under stress, either physiological or pathological, there is a decrease in variability. When pathological stresses are considered, a widespread view is to interpret this loss as a decomplexification of the cardiac system due to illness (Goldberger, 1996; Varela et al., 2010). In the domain of physiological exercise, however, a clear explanation for this phenomenon is lacking (Lewis and Short, 2010), nor is there a theory interpreting these two phenomena together. Developing a theoretical framework in this area of investigation might generate critical knowledge to understand the boundaries between health and disease, and allow knowledge translation from one domain to the other.

The first step to develop this framework consists of an extended comparison of how examples of pathological and physiological stresses affect HRV. With this in mind, the overall aim of this pilot investigation was to initiate, for the first time, a focused comparison of the patterns of change in HRV from a physiologic (i.e., exercise) and a pathologic (i.e., sepsis) stressor. The underlying hypothesis was that physiological and pathological stresses produce different effects on the body, and therefore their effects on HRV should differ. To test this hypothesis, we studied the changes in mean heart rate (HR), as well as four measures of variability belonging to different domains (Bravi et al., 2011), and associated to distinct physiological dimensions (Barrera-Ramirez et al., 2013). Each domain of variability highlights the mathematical nature of a measure of variability, while the physiological dimensions are supposed to provide a physiological rationale to the changes in variability. Therefore, by investigating those four measures, we can shed light on the values of the two classification systems. In particular, we evaluated the trends (variation over time) of those measures from two datasets, one describing healthy subjects undergoing physical exercise under dry/warm conditions, the other ambulatory patients who developed sepsis after bone marrow transplant (BMT). Through analysis of the changes between baseline variability and variability during stress (i.e., either exercise or sepsis), as well as the analysis of the correlation between the measures, we highlighted similarities and differences between the two types of stressors.

## Materials and methods

### Subjects

This study focuses on two datasets: (1) R-R interval (time between two successive R peaks during normal sinus rhythm) monitoring of 13 patients who developed systemic infection (i.e., sepsis) after BMT, and (2) R-R interval monitoring of 13 matched (by gender, age, weight, and height) healthy subjects undergoing controlled physical exercise under heat exposure, as described in more detail below. Table 1 summarizes the subject characteristics.

**Table 1:** **Demographic information**.

	**Dataset**		*p*-value^*^
	**EXERCISE (*n* = 13)**	**SEPSIS (*n* = 13)**	
Gender			
• Male, *n* (%)	9 (69%)	9 (69%)	1
• Female, *n* (%)	4 (31%)	4 (31%)	
Age [years] – Median (95% CI)	50 (29 – 62)	49 (34 – 60)	0.83
Height [cm] – Median (95% CI)	169 (166 – 178)	173 (162 – 178)	0.97
Weight [kg] – Median (95% CI)	75 (62 – 90)	76 (63 – 105)	0.79
VO_2_max [ml/kg/min] – Median (95% CI)	38.2 (31.5 – 43.2)	Not available	–

CI, Confidence Interval;

* *Wilcoxon rank-sum test was used to compare the medians, χ^2^ test was used to compare the proportions*.

### Sepsis

The first dataset (SEPSIS) consisted of patients undergoing BMT for hematological malignancy or other disorders. Inclusion criteria were treatment with myeloablative chemoradiotherapy followed by an allogeneic or autologous BMT. Exclusion criteria were pre-existing cardiopulmonary disease, taking beta-blockers or calcium-channel blockers, pre-existing arrhythmia (e.g., atrial fibrillation, atrial bigeminy), and contraindication to electrocardiogram adhesives (e.g., allergy, severe psoriasis). Sepsis was defined as the systemic inflammatory response syndrome along with a clinically suspected infection requiring treatment. Over 50% of the patients were diagnosed with sepsis based on the presence of fever, defined a priori as one recording greater than 38.5°C or two recordings greater than 38.0°C within 12 h. The remaining diagnoses were based on clinical suspicion, bacteremia, productive cough, and mucositis. For further details refer to (Ahmad et al., 2009; Bravi et al., 2012). The dataset consisted of 17 subjects: 3 who did not develop sepsis and showed an increase in HRV, 13 who developed sepsis and showed a reduction in HRV, and one insulin dependent diabetic subject who developed sepsis but showed an increase in HRV during the development of sepsis. To enable the comparison with the reduction in HRV during exercise, in this study we focused only on the 13 subjects who developed sepsis and showed a reduction in HRV. The average length of a recording was 12 days. A Zymed DigiTrak-Plus (Philips Healthcare, Canada) Holter system was used to record the R-R interval time series of each subject. Written informed consent was obtained from all participants, and the Ottawa Hospital Research Ethics Board authorized the study.

### Exercise

The second dataset (EXERCISE) included healthy normally active (i.e., untrained but not sedentary) volunteers performing intermittent exercise in warm/dry conditions. We selected 13 subjects from a pool of 73, to match the septic patients. The matching was done prior to any data analysis. The experimental protocol was approved by the University of Ottawa Health Sciences and Science Research Ethics Board. Prior to the experimental session, participants were asked to complete a Physical Activity Readiness Questionnaire (PAR-Q) to assess their eligibility to do physical activity. Maximal oxygen uptake (VO_2max_) was subsequently measured during a progressive cycle ergometer protocol which consisted of a 2-min warm-up at 40 W followed by 20 W increments every minute until the participant could no longer maintain a pedaling cadence of at least 60 rpm.

The experiments were performed at the same time of day. Participants were asked to arrive at the laboratory after eating a small breakfast and to refrain from consuming alcohol and caffeine for 24 h prior to experimentation and to avoid major thermal stimuli on their way to the laboratory. Participants were also encouraged to arrive well-hydrated as no fluid replacements were provided during the experiment. After 30 min of rest on a chair, participants performed four bouts of 15-min cycling (Corival recumbent cycle ergometer, Lode, Netherlands) at a constant rate of heat production (400 W) in an environmental chamber regulated at 35°C and 20% relative humidity. Each exercise bout was separated by 15-min of rest on the recumbent cycle, with a final 60 min recovery (not reported in the following analyses). Therefore, the length of each recording was of 150 min (60 of exercise, 90 of rest).

Electrocardiographic waveforms for each subject were recorded through a Holter DigiTrak XT (Philips Medical Systems, USA) physiological monitor. For simplicity we will refer to this dataset as EXERCISE.

### Variability computation

For both datasets, only the beats characterized as normal sinus rhythm were included, while all premature beats were excluded. The classification was automatically performed by the Holter monitors and through delta detector on both datasets (Clifford et al., 2002). Using Continuous Individualized Multiorgan Variability Analysis (CIMVA™) software (Bravi, 2013), HR and four measures of variability were extracted from the R-R interval time series of each subject through a windowed analysis, namely by using 5-min windows for both EXERCISE and SEPSIS. This means that five values were computed using 5 min of data, and successive values of those measures were computed by repeatedly shifting the 5-min window by 30 s for EXERCISE and 2.5 min for SEPSIS; these window steps were different because of the shorter length of the EXERCISE recordings compared to the SEPSIS recordings. This procedure created five variability time series per subject. Together with mean HR, the measures we investigated are standard deviation (statistical domain), the ratio between the power at low (LF) and high (HF) frequencies (LF/HF ratio—computed through the Lomb–Scargle periodogram, energetic domain), sample entropy (informational domain), and the Hurst exponent (computed through the Scaled windowed variance method, invariant domain). For details on the domains of variability, refer to Bravi et al. (2011).

### Statistical analysis

**Figure 1:**
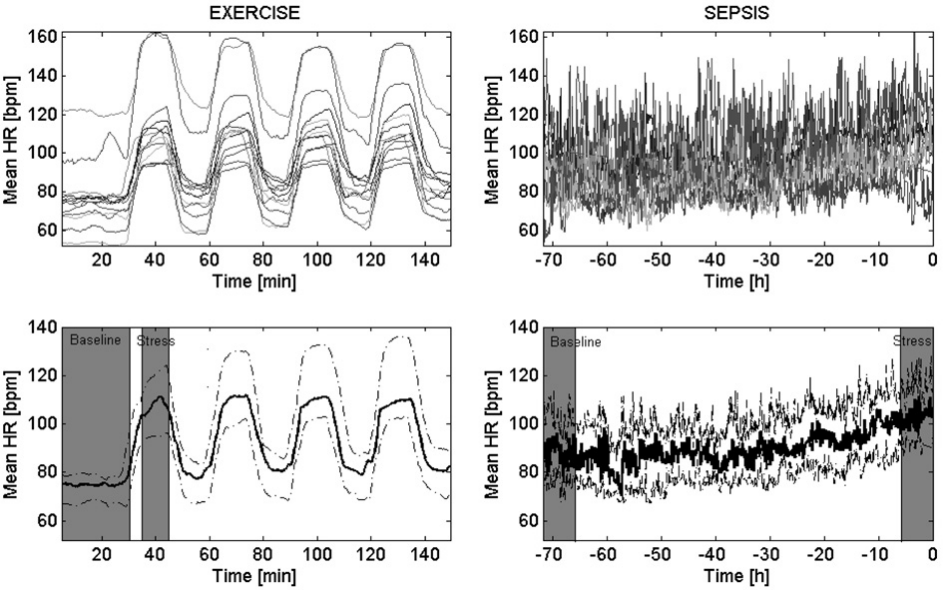
**Trends of mean heart rate in EXERCISE and SEPSIS. Top panels** show how the mean heart rate (HR) changed over time for all the subjects (above), for either EXERCISE (left) or SEPSIS (right) datasets. **Bottom panels** report the median trends across each population—i.e., population trend—(bold solid line), together with the 95% confidence interval (dashed line). The EXERCISE time series start from 30 min prior the beginning of the first exercise bout, to the end of the last resting period. The SEPSIS time series start from 72 h prior the administration of antibiotics, to the time of administration of antibiotics (*t* = 0). In gray are highlighted the areas we referred to as “baseline” and “stress.”

Before any analysis, each of the time series for the patients in the SEPSIS dataset were aligned with respect to the time of administration of antibiotics, and the time series for the subjects in the EXERCISE dataset were aligned with respect to the start of the exercise routine. Following the alignment, three analyses were performed. (1) Qualitative inspection of the population trends of HR and the four HRV measures (reported as median and 95% confidence intervals around the median). The population trends were computed by taking at each time instant the median value across the different subjects, as show in Figure 1. This led to five population trends for each dataset. (2) Statistical evaluation of the change between variability at baseline, and variability during stress. First, the variability time series were segmented, as summarized in Table 2, and then for each subject the median variability over time was computed, creating a distribution of values representative of either baseline or stress, for both SEPSIS and EXERCISE. The Wilcoxon sign-rank test was used to evaluate the null hypothesis of zero change between baseline and stress. It is worth mentioning that what we defined as “variability at baseline” for the SEPSIS dataset is not really representative of baseline conditions, because likely some subjects were already developing sepsis 72 h before the administration of antibiotics. However, not all the subjects had more than 72 h of data prior the administration of antibiotics, therefore to include all of them we decided to use that time interval. (3) Spearman's non-linear correlation analysis across the five population trends of SEPSIS and EXERCISE during the time intervals representative of “stress,” as specified in Table 2.

**Table 2:** **Segmentation of the time series**.

**Dataset**	**Baseline**			**Stress**		
	**Start**	**End**	**Length**	**Start**	**End**	**Length**
EXERCISE	Thirty minutes before the beginning of the first^*^ exercise bout	At the beginning of the first^*^ exercise bout	30 min	Ten minutes before the end of the first exercise bout	At the end of the first exercise bout	10 min
SEPSIS	Seventy-two hours prior the administration of antibiotics	Sixty-six hours prior the administration of antibiotics	6 h	Six hours prior the administration of antibiotics	At the time of administration of antibiotics	6 h

**Figure 2:**
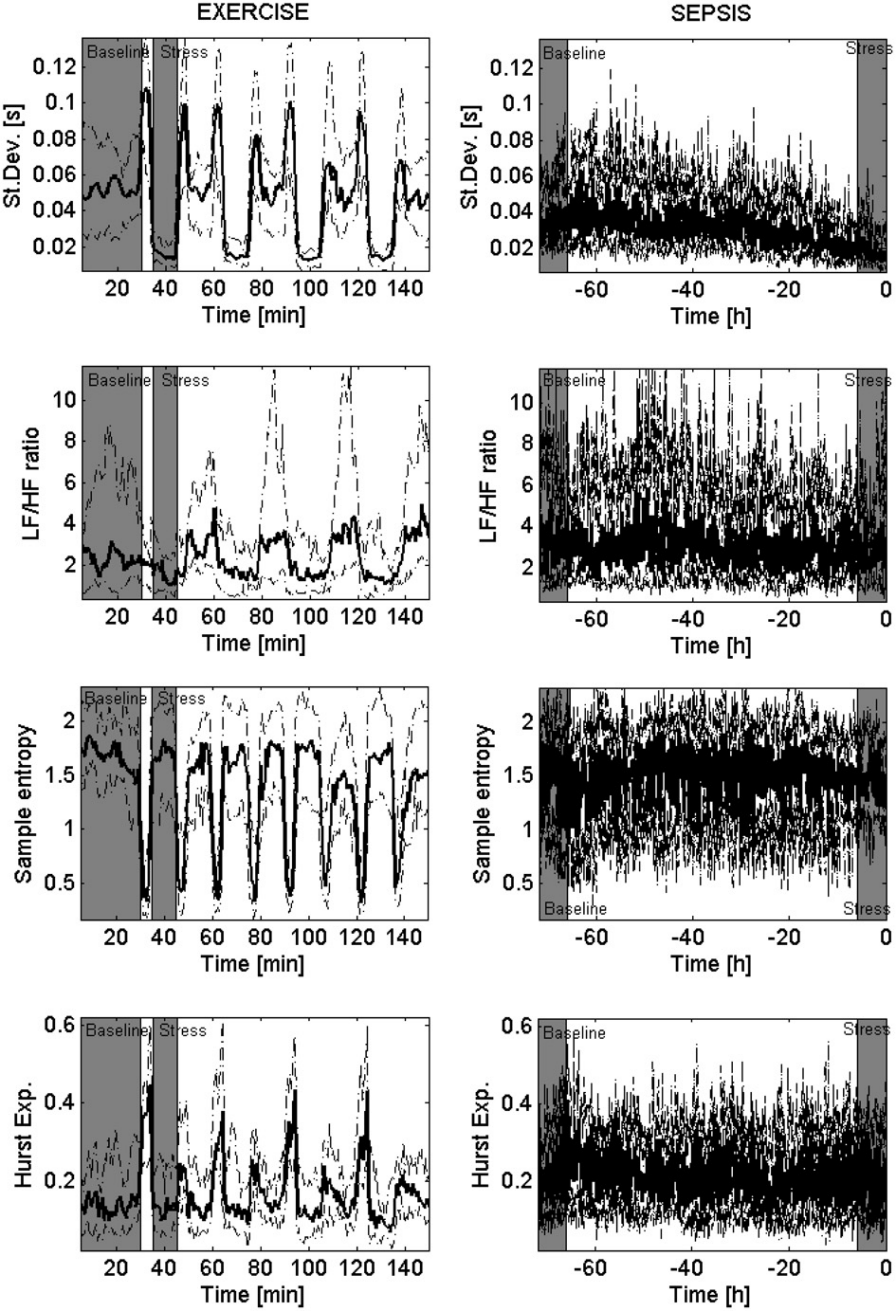
**Population trends in physiological dimensions of variability**. These eight panels show the population trends of four measures of variability, hypothetically linked to separate physiological dimensions. From the top, each row presents: standard deviation, LF/HF ratio (computed through the Lomb–Scargle periodogram), sample entropy, and Hurst exponent (computed through Scaled windowed variance). The columns depict the median trend across all the subjects (bold solid line) and its 95% confidence intervals (dashed line) for the EXERCISE dataset (left) and the SEPSIS dataset (right). In gray are highlighted the areas we referred to as “baseline” and “stress.”

*This table shows the time intervals that were used to compare the changes in HRV between baseline condition and stress. The variability time series were segmented as specified, and the median value in each time interval was computed. This procedure created two values (i.e., at baseline and during stress) for each subject and for each variability time series. Those intervals are reported in Figures 1, 2 as gray areas*.

* *Different exercise bouts did not produce significant changes in the results*.

## Results

The alignment of the variability time series of each subject, as well as the creation of the population trends of the mean HR are reported in Figure 1. The figure shows that the mean HR considerably increased during exercise, and slightly increased during sepsis. Because of the low inter-subject variability, the 95% confidence intervals around the population trends are tight. Similarly, Figure 2 shows the population trends for standard deviation, LF/HF ratio, sample entropy and Hurst exponent. Standard deviation presented a clear population trend in both sepsis and exercise, both in terms of a decrease in time as well as a tightening of the confidence intervals. The same phenomenon was not observed in either sample entropy nor Hurst exponent, even though they showed like standard deviation, a high sensitivity to non-stationarity (i.e., spikes in the transition between exercise and rest, and vice versa). Lastly, the LF/HF ratio showed a slight reduction in only the EXERCISE population trend, supported by a considerable reduction of the 95% confidence intervals around the population trends.

**Figure 3:**
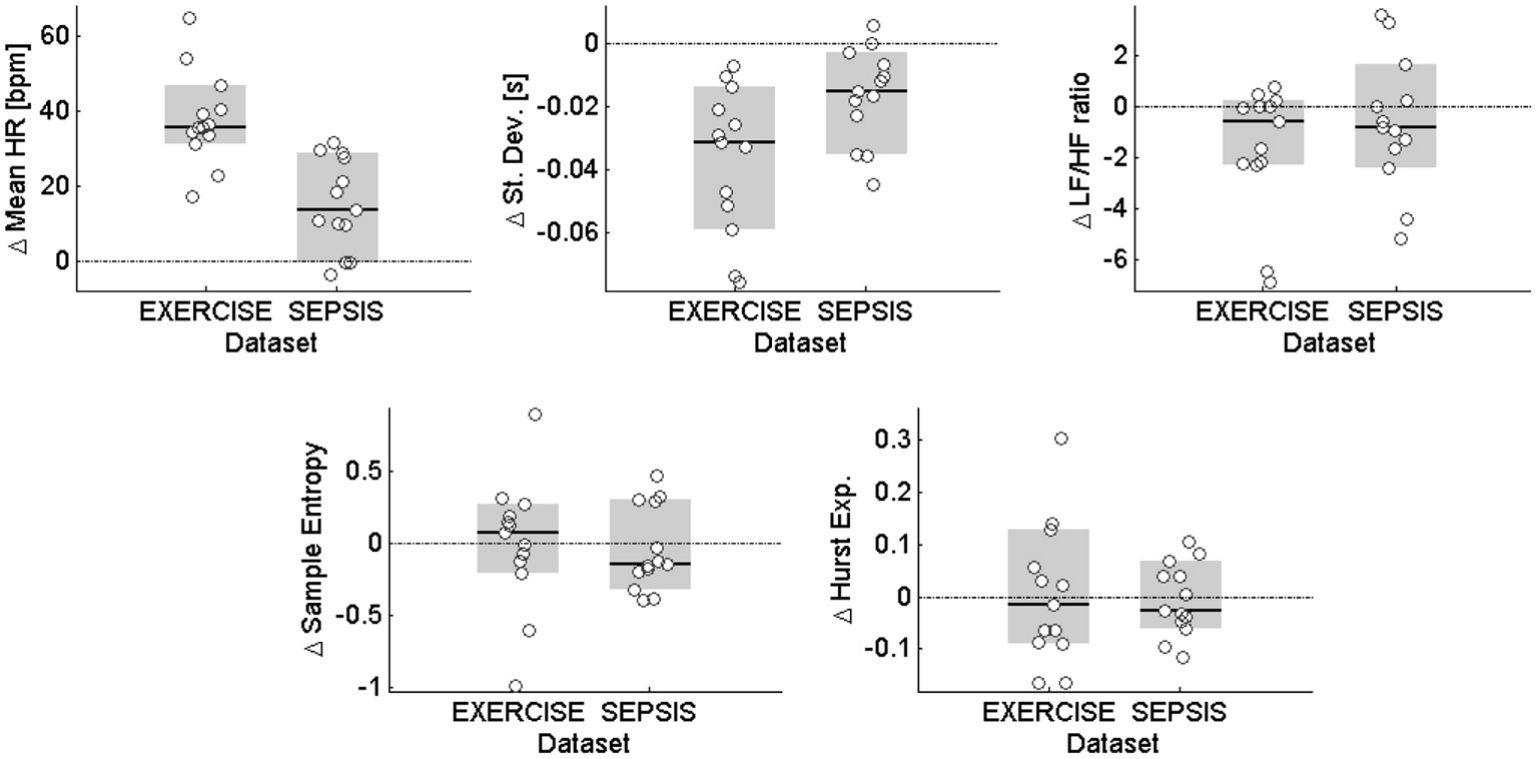
**Distributions of change from baseline to stress**. The five panels show the distributions for mean HR and the four investigated measures of HRV. Each white circle represents the change in the median variability of a given subject from baseline to stress, as described in Table 3. The black solid line is the median of the dataset, and the gray area identifies the 95% confidence intervals for the median. The horizontal dash-dot lines highlight the lines of no change from baseline to stress.

To evaluate the changes between baseline and stress in a quantitative way, the values of the measures (median and 95% confidence intervals), together with results of the Wilcoxon sign-rank test are reported in Table 3. Figure 3 provides a visual representation of the distributions of change from baseline to stress of Table 3. Mean HR and standard deviation confirmed the changes observed from a visual inspection, rejecting the null hypothesis of no change in the transition from baseline to stress for both EXERCISE (*p* = 0.0002) and SEPSIS (*p* = 0.003). The LF/HF ratio rejected the null hypothesis only in EXERCISE (*p* = 0.04). Hurst exponent and sample entropy were not able to reject the null hypothesis in neither EXERCISE nor SEPSIS.

**Table 3:** **Statistical results**.

**Measure**	**EXERCISE**				**SEPSIS**			
	**Baseline**	**Stress**	**Change**	***p*-value**	**Baseline**	**Stress**	**Change**	***p*-value**
Mean heart rate	74	110	36	**0.0002**	82	99	13	**0.003**
	(68, 78)	(95, 121)	(31 47)		(79, 98)	(92, 114)	(−0.2 29)		
Standard deviation	0.050	0.014	−0.031	**0.0002**	0.032	0.016	−0.015	**0.002**
	(0.026, 0.076)	(0.010, 0.021)	(−0.059, −0.013)		(0.023, 0.044)	(0.013, 0.028)	(−0.035, −0.002)	
LF/HF ratio	2.41	1.09	−0.63	**0.04**	2.94	2.58	−0.82	0.30
	(0.99, 5.77)	(0.81, 2.36)	(−2.31, 0.23)		(1.92, 6.18)	(1.50, 4.56)	(−2.44, 1.61)	
Sample entropy	1.67	1.79	0.08	0.73	1.57	1.43	−0.14	0.63
	(1.53, 1.94)	(1.17, 2.13)	(−0.21, 0.27)		(1.24, 1.71)	(1.25, 1.56)	(−0.32, 0.30)	
Hurst exponent	0.15	0.13	−0.01	0.79	0.21	0.19	−0.03	0.83
	(0.08, 0.24)	(0.07, 0.24)	(−0.09, 0.13)		(0.16, 0.25)	(0.14, 0.25)	(−0.06, 0.07)		

*This table shows the values for each measure after the segmentation specified in Table 2, together with the Change (Stress minus Baseline, within each subject). Since baseline and stress were computed from repeated measurements on the same subjects, the p-values were computed through Wilcoxon sing-rank test. In bold are reported significant rejections of the null hypothesis of zero change (α-value = 0.05)*.

The final analysis compared the degree of correlation between the population trends at the time of stress. The results are summarized in Table 4. Mean HR and standard deviation showed a good correlation (−0.68) during exercise, but lower correlation during sepsis (−0.40). Similarly, the levels of correlation between mean HR, standard deviation, LF/HF ratio and sample entropy resulted above |0.5| during exercise and below |0.5| during sepsis. The Hurst exponent showed low levels of correlation with all the measures in both sepsis and exercise, exception made for the correlation with standard deviation, which reached 0.51 during sepsis.

**Table 4:** **Spearman correlation matrix of the population trends in EXERCISE and SEPSIS, during stress**.

	**Mean HR**	**Standard deviation**	**LF/HF ratio**	**Sample entropy**	**Hurst exponent**
Mean HR	1.00, **1.00**				
Standard deviation	−0.68, **−0.40**	1.00, **1.00**			
LF/HF ratio	−0.76, **0.19**	0.71, **−0.17**	1.00, **1.00**		
Sample entropy	0.78, **−0.13**	−0.57, **0.05**	−0.58, **−0.14**	1.00, **1.00**	
Hurst exponent	−0.34, **−0.24**	0.22, **0.51**	−0.13, **−0.15**	−0.33, **−0.13**	1.00, **1.00**

*This table shows the correlation values between the population time series in the two datasets, making use of only those samples representative of the stress phase (as specified in Table 2). The coefficients for SEPSIS are reported in bold, while the others are for EXERCISE*.

## Discussion

The main objective of this study was to explore the differences between changes in variability due to exercise vs. sepsis; the underlying hypothesis being that the two types of stress produce differential impact on HRV measures.

Before interpreting the results, it is essential to highlight at a higher level what each measure of variability represents. It is well-established that despite the differences from a mathematical point of view, many (but not all) measures of variability are highly correlated with each other (Maestri et al., 2007), and are sensitive to different types of stressors affecting the cardiovascular system; this is compatible with our findings. Therefore, we believe that each measure of variability can be interpreted as a non-specific sensor, which may be sensitive to multiple physiological mechanisms.

HRV demonstrated two main behaviors, possibly associated with two specific types of physiological phenomena: (1) sensitivity to exercise only (with “sensitivity” we intend the ability to show a change between baseline and stress—as shown in Table 3), or (2) to both sepsis and exercise. Although a loss of complexity might have been expected with sepsis development as opposed to exercise, this was not observed. Furthermore, we repeated the same analyses described in this article with the 11 HRV measures found to be relevant in tracking sepsis development in a previous study making use of the same SEPSIS dataset (Bravi et al., 2012), and we identified that all those measures changed significantly in both SEPSIS and EXERCISE (results not shown). This highlights that HRV responds similarly to both sepsis and exercise, although with differences, as exemplified by the particular behavior of the LF/HF ratio. This is similar to what was seen in a subsequent analysis on a set of other 17 measures of HRV, out of a pool of 96 measures (results not shown). Furthermore, when multiple measures of variability are compared through multivariate time series analysis (as done in Table 4 through correlation analysis), further differences between sepsis and exercise arise. This result supports the idea that the understanding of the physiology underlying variability may require the integration of information from multiple HRV measures.

The similarities between sepsis and exercise could be explained based on physiology. Exercise-induced immune responses and inflammatory-related immune responses share similar physiological mechanisms, such as the release of cytokines and mediators, albeit with a different intensity (Shephard, 2001; Bente Klarlund, 2005). However, elevated levels of physical stress, such as those associated with performing exercise at very high intensity (=90% of VO_2_peak), produce a much lower immune response compared to the septic one (Shephard, 2001). Because we saw major changes during exercise rather than sepsis for both mean HR and standard deviation (Table 3), it is unlikely that immune response played a major role on the observed changes in HRV. Septic patients tend to show a normal or high cardiac output, due to an increased HR, a reduced afterload (due to the reduced vascular tone), and either increased (due to catecholamines) or reduced contractility (myocardial depression mediated by inflammatory cytokines) (Vincent, 2008). These similarities in the cardiovascular response could justify the similar behavior observed during sepsis and exercise, highlighting that major changes in HRV are likely driven by pure cardiovascular regulation.

**Figure A1:**
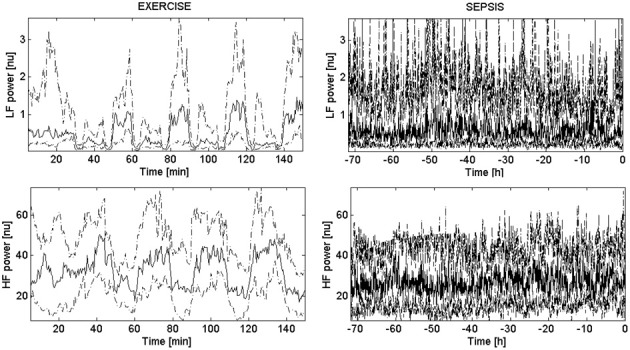
**Population trends of LF and HF power**. Body: These four panels show the population trends of LF (top) and HF (bottom) power computed through the Lomb–Scargle periodogram. Each panel depicts the median trend across all the subjects (bold solid line) and its 95% confidence intervals (dashed line) for the EXERCISE dataset (left) and the SEPSIS dataset (right).

On the other hand, the particular behavior of the LF/HF ratio is more difficult to explain, especially given the fact that this measure was documented to be sensitive to sepsis in multiple pilot studies, as specified in a recent review (Buchan et al., 2012). The key difference with the literature is that we did not compare septic patients with controls, but rather evaluated the change of LF/HF ratio during sepsis development. For instance, a study on 81 patients (Chen and Kuo, 2007) showed that septic patients (*n* = 21) who subsequently developed shock had lower LF/HF ratio respect to patients who did not develop sepsis (*n* = 60). In our study the LF/HF ratio showed a reduction during sepsis development (Table 3) which was not statistically significant. However, the three patients which we excluded from the analysis because did not develop sepsis showed instead an average LF/HF ratio of 3.3 (CI: 3.0, 3.6) during stress, showing therefore a larger average LF/HF ratio (see Table 3), consistent with the literature. Although the lack of control of posture imperils our ability to further interpret the LF/HF ratio, it is of interest that this measure exhibited different behavior during sepsis and exercise. For future reference, Figure A1 in Appendix shows the details of LF and HF power (in normalized units) for both EXERCISE and SEPSIS, highlighting the major contribution of LF power in the LF/HF ratio population shown for EXERCISE (Figure 2).

An outcome of our analyses is that HRV measures taken from different domains of variability are likely to bring “unique” pieces of information, as shown by their low level of correlation (Table 4). The physiological interpretation of each measure in lights of the physiological dimensions of (Barrera-Ramirez et al., 2013) is instead more challenging. Standard deviation decreased during both sepsis and exercise, possibly confirming the hypothesis that it represents the level of cardiopulmonary reserve of the body (i.e., the lowest the measure, the more the body is toward its maximal capacity). LF/HF ratio, measure of sympathovagal modulation, was reduced during exercise, rather than increasing as shown in Barrera-Ramirez et al. (2013). Furthermore, despite the increase in HR, no significant change of LF/HF was detected during sepsis. Sample entropy, being a measure of complexity, was supposed to be primarily affected by a state of illness; despite the expectations, no change was observed during sepsis development. Lastly, the Hurst exponent has been linked to the capacity of the cardiorespiratory system to deliver oxygen and clear carbon dioxide. Given the increased demand of oxygen during exercise, we would have expected to see a decrease in the measure (*H* = 0.5 indicates no fractality), which however did not appear.

### Limitations

While a wider analysis using additional measures of variability is possible, we are cognizant of the small size of the patient populations, and wish not to over-analyze nor over-interpret the results. With a larger dataset in the future, it may be possible to better relate changes in HRV to the current theories about the nature of variability in physiological systems (Seely and Macklem, 2012; Barrera-Ramirez et al., 2013). It is worth noticing also the need to characterize the sensitivity of each measure of variability to non-stationarity. As shown in Figure 2, standard deviation, sample entropy and Hurst exponent showed significant sensitivity to the transitions between rest and exercise (and vice versa). This was not a problem for the data analysis, given we knew the acquisition protocol; however, if we imagine the monitoring of ambulatory patients, the necessity of distinguishing non-stationarity from clinically relevant changes in HRV becomes clear.

An inherent limitation to comparing these two cohorts is the lack of standardization of the severity of the stressor, particularly true for the subjects developing sepsis. Indeed, most of the subjects in that group where diagnosed based on fever and clinical impression, possibly introducing a confounding factor in the analysis. Further work on the definition of what “stress” is and how to quantify it would enable a better comparison of different physiologic and pathologic forms of stress. On the same line, the choice of the specific type of physiologic and pathologic stress is a limiting factor of the analyses, and the investigation of alternative protocols or pathologies would be of interest to validate the results. Another limitation is that, the patients in the two populations were not matched by fitness level (e.g., VO_2_max), because that information was unavailable for the septic population. Given the similar changes seen in univariate HRV in both populations, it is likely that the fitness level did not produce a bias in the analysis. A similar argument could be applied to other systematic differences between the two datasets, e.g., different definition of baseline state or degree of intensity of the stressor. Also, our results are based on a set of arbitrary choices determined *a priori*, e.g., chosen window size, measures of variability, their computational parameters, time segments for the definition of baseline and stress (i.e., Table 2), and use of the median to create the population trend. A larger dataset would enable a sensitivity analysis respect to those parameters. Despite these limitations, this work represents an initial step toward the definition of the differences between physiological and pathological stressors, and the understanding of the physiological mechanisms underlying HRV.

## Conclusions

Qualitative and quantitative comparisons of the changes in HRV during physiological and pathological stress were performed on two datasets, one from patients who developed sepsis after a BMT (pathological stress), the other from a matched group of healthy subjects performing physical exercise (physiological stress). The comparison showed that HRV measures behave similarly during both exercise and sepsis development, although with subtle differences, whose explanation remains fertile ground for further investigation.

## Author contributions

Andrea Bravi, Andrew J. E. Seely, and André Longtin conception and design of research; Andrea Bravi analyzed data; Andrea Bravi, Andrew J. E. Seely, André Longtin, Christophe Herry, Geoffrey Green, Glen P. Kenny interpreted results; Andrea Bravi, Andrew J. E. Seely, André Longtin, Christophe Herry, Geoffrey Green, Glen P. Kenny, Heather E. Wright edited and revised manuscript; Andrea Bravi, Andrew J. E. Seely, André Longtin, Christophe Herry, Geoffrey Green, Glen P. Kenny, Heather E. Wright approved final version of manuscript.

### Conflict of interest statement

Andrew J. E. Seely is founder and Chief Science Officer of Therapeutic Monitoring Systems, Inc. (TMS), created to commercialize patented Continuous Individualized Multi-organ Variability Analysis (CIMVA) technology, with the objective of delivering variability-directed clinical decision support to improve quality and efficiency of care. Geoffrey Green is currently employed by TMS in the position of Product Manager. The other authors declare that the research was conducted in the absence of any commercial or financial relationships that could be construed as a potential conflict of interest.
